# Apolar Compounds in Seaweeds from Fernando de Noronha Archipelago (Northeastern Coast of Brazil)

**DOI:** 10.1155/2012/431954

**Published:** 2012-01-09

**Authors:** Leandro De Santis Ferreira, Izabel Cristina Casanova Turatti, Norberto Peporine Lopes, Thais Guaratini, Pio Colepicolo, Eurico Cabral Oliveira Filho, Ricardo Clapis Garla

**Affiliations:** ^1^Departamentos de Física e Química, Faculdade de Ciências Farmacêuticas de Ribeirão Preto, Universidade de São Paulo, 14040-903 Ribeirão Preto, SP, Brazil; ^2^Lychnoflora Pesquisa e Desenvolvimento em Produtos Naturais LTDA, Incubadora SUPERA, Campus da USP, 14040-900 Ribeirão Preto, SP, Brazil; ^3^Instituto de Química, Universidade de São Paulo, 05513-970 São Paulo, SP, Brazil; ^4^Instituto de Biociências, Universidade de São Paulo, P.O. Box 11461, 05508-090 São Paulo, SP, Brazil; ^5^Departamento de Oceanografia e Limnologia, Centro de Biociências, Universidade Federal do Rio Grande do Norte, 59072-970 Natal, RN, Brazil

## Abstract

Hyphenated techniques of gas chromatography coupled to mass spectrometer were used to determine fatty acids in eleven species of seaweeds from Fernando de Noronha archipelago. The main compounds detected in all studied species were the alcohol phytol and the fatty acids 14 : 0; 15 : 0; 16 : 0; 18 : 0; 18 : 1 n^9^; 18 : 2 Δ^9,12^; 20 : 4; 20 : 5. These fatty acids are commonly found in seaweeds present in warm regions. Thus, we found no specificity in the presence of a particular set of fatty acids and the studied species indicating that they are not useful as taxonomic indicators. However, they could be used in a comparative study with algae found in polluted area because many of the studied seaweeds are widespread and Fernando de Noronha has low human influence.

## 1. Introduction

Seaweeds are key ecological factors in shallow marine areas forming the base of the trophic web and structuring ecosystems especially on consolidated substrata. They are known by the production of many bioactive compounds. Hence, the pharmaceutical and the cosmetics industries have a special interest in algae as sources of specific molecules [1–4]. Also, several works have investigated their value in human and animal nutrition [5–8]. Among the algae-derived compounds, polyunsaturated fatty acids are especially important as they act as antioxidant agents involved in many physiological processes [9, 10].

The studies to determine the fatty acids profiles started some years before and many of them focused on the use of these compounds as biomarkers for chemotaxonomy, though their concentration may be susceptible to environmental interference [11–14]. Some of the main important factors that influence the algae fatty acid concentration are the temperature, [15, 16], types of habitat [15, 17], and presence of metals and pollutants [12, 13, 18]. Although less common, other approaches using fatty acids have explored the effects of industrial effluents and environmental variables on the amount and quality of fatty acids produced [19, 20] and their role in food assimilation by herbivorous invertebrates [21].

Fernando de Noronha archipelago is an isolated group of islands formed by relatively recent volcanic processes. The islands are located approximately 350 km off the northeastern Brazilian coast and are part of Pernambuco State [22]. The archipelago was established as a marine protected area since 1988, and its marine flora is composed of 128 taxa, including 44 species of Chlorophyta, 62 of Rhodophyta, and 22 of Phaeophyta [23]. The most abundant benthic algae groups found in the archipelago are represented by the families Dictyotaceae and Sargassaceae. Other seaweeds also commonly found are the green algae *Caulerpa verticillata* and red algae *Galaxaura* spp. [23, 24]. As part of a broader research project aimed to study chemical compounds of seaweeds from Fernando de Noronha, herein is provided baseline information on the fatty acids produced by eleven species commonly found in the archipelago.

## 2. Experimental

### 2.1. Field Collection

A list of the studied species is presented in Table 1. Samples were collected between February and March 2006 at two sites of the main island, Caieiras Beach (3°50′18.8′′S, 32°23′57.3′′W) and Sueste Bay (3°52′1.2′′S, 32°25′19.7′′W). Research permit from the Brazilian Environmental Agency (IBAMA) to collect algae was registered under number 050/2006. Seaweeds were randomly collected by hand by uprooting the whole plant, which were placed in labeled plastic bags, frozen, and sent to the laboratory. The algae were identified, cleaned from epiphytes, animals, and sediment, washed with distilled water, and in an oven at 40°C dried for seven days.

### 2.2. Preparation of Samples

Ten milliliters (10 mL) of dichloromethane (J. T. Baker, Phillipsburg, NJ, USA) were added to 1 g (dried weight) of each species of algae and submitted to ultrasonic bath for 30 minutes. This procedure was repeated three times and the total extract was concentrated on nitrogen gas. Subsequently, 2.0 mL of 1.0 M sodium methoxide were added to the extract and shaken occasionally during five minutes at 65°C. After cooling the extract, 1.0 mL of water and three samples of 1.0 mL of chloroform (J. T. Baker, Phillipsburg, NJ, USA) was added to each sample, shaken for one minute, and centrifuged at 3000 rpm, or 1612.8 g, to extract methyl esters of the fatty acids. The chloroform phase (3.0 mL), was removed and nitrogen gas was used to evaporate the solvent. Samples were suspended in 1.0 mL ethyl acetate (J. T. Baker, Phillipsburg, NJ, USA), and sodium sulphate anhydrous (Sigma Inc., St. Louis, MO, USA) was added to remove water. This methodology was adapted from Eder et al. [25, 26].

### 2.3. GC-MS Analysis

Samples were analyzed through GC-MS (gas chromatography coupled to mass spectrometer detector) in a Shimadzu QP2010 with ionization source of 70 eV and fragmentation by electronic ionization (EI). The volume of 1.0 *μ*L for each sample was injected at 220°C in a DBWAX column (30 m × 0.25 mm × 0.25 *μ*m). The analysis occurred with 1-minute sample time in the splitless mode, a column flow of 1.3 mL min^−1^, a linear velocity of 41.4 cm s^−1^, and scan between *m/z* 40 and *m/z* 500. Oven's temperature started with 50°C, increasing to 20°C min^−1^ until 200°C, kept in this temperature for 5 minutes, then increased to 5°C min^−1^ until 230°C and kept in this temperature for 30 minutes. For identification of compounds, the peaks were compared with some standards and always consulting the libraries WILEY version no. 7 and NIST version nos. 12 and 62.

## 3. Results and Discussion

The GC-MS analysis had excellent resolution, and the characteristics of the detected unsaturated and saturated fatty acids are given in Tables 2 and 3. The eleven species have myristic acid (14 : 0), pentadecanoic acid (15 : 0), palmitic acid (16 : 0), stearic acid (18 : 0), oleic acid (18 : 1, n^9^), linoleic acid (18 : 2 Δ^9,12^), arachidonic acid (20 : 4), and eicosapentaenoic acid (20 : 5). The major compounds were hexadecanoic acid methyl ester and phytol, which were detected in all samples analyzed. Other apolar compounds, mainly alcohols, were also identified and are provided in Table 4.

The only seaweed in which 20 : 0 fatty acid was absent was the green algae, *C. verticillata*, and the acid 17 : 0 was absent in only one red algae. There were problems in isomer identification while conducting tentative taxonomic analysis, especially in double and triple bonds of C16 and C18 fatty acids, due to great similarity in retention time and in the molecular fragmentation in mass spectrum.

All compounds detected are common in macroalgae [14]. Few differences were observed in the production of fatty acids by brown, green, and red algae. Also, as only one species of green seaweed was analyzed, this prevents further taxonomic comparisons with brown and red algae. In spite of the fact that GC-MS is a highly efficient and sensitive method of analysis, it was unable to precisely determine which of the polyunsaturated fatty acids with 16 or 18 atoms of carbon was present in each species. Other apolar compounds detected had a more restrict distribution among species, and although most of them were detected in only one species, it was not possible to use them as chemotaxonomy markers, that is, phytol or loliolide. Moreover, the fatty acids referred to as chemotaxonomic agents in previous investigations [14, 17] did not allow discerning brown, green, and red algae in the present study.

Fernando de Noronha is an area subjected to low levels of pollution because it is a marine protected area where anthropic activities are controlled. Hence, it would be interesting to compare the fatty acid profiles obtained in this study with the ones from coastal areas of the main land near of the archipelago region that has different degrees of pollution. For example, *Sargassum *spp. fatty acid profiles or other algae species, which are widely distributed and resistant to environmental changes [20], might be compared to the profiles of specimens collected in areas subjected to agrotoxics, solvents, and metals, as already done by Tewari et al. [19] with mercury. In addition, the baseline information obtained may also be applicable to a long-term ongoing research focusing on the sea urchins, which are important grazers in the archipelago [27], in order to determine possible relationships between the algae species consumed and their assimilation efficiency by those invertebrates.

## 4. Conclusion

The methodology used allowed determining the fatty acid profile of eleven seaweed species from Fernando de Noronha archipelago. As no specificity was found in the presence of a particular set of fatty acids, these compounds could not be used as taxonomic indicators in this case.

##  Conflict of Interests

The authors declare that do not have any conflict of interests.

## Figures and Tables

**Table 1:** Classification for the eleven species of seaweeds from Fernando de Noronha archipelago in northeastern Brazil analyzed in this study.

Divisions and species
Chlorophyta
*Caulerpa verticillata* J. Agardh, 1847
Rhodophyta
*Asparagopsis taxiformis *(Delile) Trevisan de Saint-Léon, 1845
*Dictyurus occidentalis* J. Agardh, 1847
*Dichotomaria marginata *(J. Ellis & Solander) Lamark, 1816
*Dichotomaria obtusata* (J. Ellis & Solander) Lamarck, 1816
*Galaxaura rugosa *(J. Ellis & Solander) J. V. Lamouroux, 1816
Ochrophyta
*Dictyota cervicornis* Kützing, 1859
*Dictyopteris justii* J. V. Lamouroux, 1809
*Dictyopteris plagiogramma *(Montagne) Vickers, 1905
*Padina gymnospora* (Kützing) Sonder, 1871
*Sargassum *sp.

**Table 2:** Saturated fatty acids present in the eleven seaweeds studied.

Fatty Acids	At	Gr	Db	Dm	Do	Cv	Dc	Dj	S	Pg	Dp
12 : 0	−	−	−	−	−	+	+	+	−	−	−
14 : 0	+	+	+	+	+	+	+	+	+	+	+
15 : 0	+	+	+	+	+	+	+	+	+	+	+
16 : 0	+	+	+	+	+	+	+	+	+	+	+
17 : 0	+	+	−	+	+	+	+	+	+	+	+
18 : 0	+	+	+	+	+	+	+	+	+	+	+
20 : 0	+	+	+	+	+	−	+	+	+	+	+
22 : 0	+	+	+	+	−	−	−	+	+	+	+
24 : 0	+	+	+	+	−	+	−	+	+	−	−

+ presence; − absence. At :  *Asparagopsis taxiformis*; Gr :  *Galaxaura rugosa*; Db :  *Dichotomaria obtusata*; Dm :  *Dichotomaria marginata*; Do :  *Dictyurus occidentalis*; Cv :  *Caulerpa verticillata*; Dc :  *Dictyota cervicornis*; Dj :  *Dictyopteris justii*; S :  *Sargassum *sp.; Pg :  *Padina gymnospora*; Dp :  *Dictyopteris plagiogramma*.

**Table 3:** Unsaturated fatty acids founded in the eleven seaweeds studied.

Fatty Acids	At	Gr	Db	Dm	Do	Cv	Dc	Dj	S	Pg	Dp
14 : 1 n^5^	−	−	−	−	−	+	−	−	−	−	−
16 : 1 n^7^	+	+	+	−	+	+	+	+	+	−	−
16 : 1 n^9^	+	+	−	−	+	+	+	+	+	+	−
16 : 1 n^9^ ETHYL	−	−	−	−	+	−	−	−	−	−	−
16 : 1 n^11^ ^or^ ^13^	+	−	−	−	+	+	+	+	+	+	−
16 : 2 Δ^7,10^ ^or^ ^9,12^	+	−	−	−	−	+	−	−	−	−	−
16 : 3 Δ^4,7,10^	−	−	−	−	−	−	−	+	+	+	+
18 : 1 n^9^	+	+	+	+	+	+	+	+	+	+	+
18 : 1 n^10^ ^or^ ^11^ ^or^ ^12^	+	+	+	+	+	+	+	+	+	−	+
18 : 1 n^13^ ^or^ ^14^ ^or^ ^16^	+	−	−	−	+	+	+	+	−	+	−
18 : 2 Δ^8,11^	−	−	−	−	−	−	−	+	−	−	−
18 : 2 Δ^9,12^(E,E) or (Z,Z)	+	+	+	+	+	+	+	+	+	+	+
18 : 3 Δ^6,9,12^	+	−	−	−	+	+	−	+	+	+	−
18 : 3 Δ^9,12,15^	+	+	+	−	+	+	+	+	+	+	−
20 : 1 n^9^ ^or^ ^ 11^	−	+	−	−	−	−	−	−	+	+	−
20 : 2 Δ^11,14^	+	+	−	−	−	+	−	+	+	+	−
20 : 3 Δ^7,10,13^ ^or^ ^ 8,11,14^	+	−	−	−	+	+	+	+	+	+	−
20 : 4	+	+	+	+	+	+	+	+	+	+	+
20 : 5	+	+	+	+	+	+	+	+	+	+	+
22 : 1 n^13^	+	−	−	−	−	−	−	−	+	−	−

+ presence; − absence. At: *Asparagopsis taxiformis*; Gr: *Galaxaura rugosa*; Db: *Dichotomaria obtusata*; Dm: *Dichotomaria marginata*; Do: *Dictyurus occidentalis*; Cv: *Caulerpa verticillata*; Dc: *Dictyota cervicornis*; Dj: *Dictyopteris justii*; S: *Sargassum sp*.; Pg: *Padina gymnospora*; Dp: *Dictyopteris plagiogramma*.

**Table 4:** Other apolar compounds found in seaweed species from Fernando de Noronha.

Compound	At	Gr	Db	Dm	Do	Cv	Dc	Dj	S	Pg	Dp
1-Tetradecanol	−	−	−	+	−	−	−	−	−	−	−
1-Hexadecanol	+	+	+	+	−	−	−	−	+	−	+
1-Hexadecanol-2-methyl	−	−	+	−	−	−	−	−	−	−	−
1-Pentadecanol	−	−	−	+	−	−	−	−	−	−	−
1-Octadecanol	+	+	+	+	−	−	−	−	+	−	+
Phytol	+	+	+	+	+	+	+	+	+	+	+
Hexadecane, 1-iodo	+	−	−	−	−	−	−	−	−	−	−
Benzeneacetamide, N-aminocarbonyl	+	−	−	−	−	−	−	−	−	−	−
3-Buten-2-one, 4-(4-hydroxy-2,2,6-trimethyl-7-oxabicyclo[4.1.0]hept-1-yl)	−	+	+	+	−	−	−	−	+	−	+
Tetrapentacontan,1,54-dibromo	−	−	−	−	−	−	−	−	+	−	−
3-Octyl oxiraneoctanoic acid methyl ester	−	−	−	−	−	−	−	+	−	−	−
1-Docosanol	+	−	−	−	−	−	−	−	−	−	−
Hexadecanamide	+	−	−	−	−	−	−	−	−	−	−
Loliolide	+	+	+	+	−	−	−	−	+	−	+
Isololiolide	+	+	+	+	−	−	−	−	+	−	+

+ presence; − absence. At: *Asparagopsis taxiformis*; Gr: *Galaxaura rugosa*; Db: *Dichotomaria obtusata*; Dm: *Dichotomaria marginata*; Do: *Dictyurus occidentalis*; Cv: *Caulerpa verticillata*; Dc: *Dictyota cervicornis*; Dj: *Dictyopteris justii*; S: *Sargassum *sp.; Pg: *Padina gymnospora*; Dp: *Dictyopteris plagiogramma*.
